# Recognition of Schizophrenia with Regularized Support Vector Machine and Sequential Region of Interest Selection using Structural Magnetic Resonance Imaging

**DOI:** 10.1038/s41598-018-32290-9

**Published:** 2018-09-14

**Authors:** Rowena Chin, Alex Xiaobin You, Fanwen Meng, Juan Zhou, Kang Sim

**Affiliations:** 1 Research Division, Institute of Mental Health, Singapore, 10 Buangkok View, Singapore, 539747 Singapore; 2 Health Services & Outcomes Research, National Healthcare Group, 3 Fusionopolis Link, Singapore, 138543 Singapore; 3 Neuroscience & Behavioral Disorders Program, Duke-NUS Medical School, 8 College Road, Singapore, 169857 Singapore; 4 West Region, Institute of Mental Health/Woodbridge Hospital, Singapore, 10 Buangkok View, Singapore, 539747 Singapore

## Abstract

Structural brain abnormalities in schizophrenia have been well characterized with the application of univariate methods to magnetic resonance imaging (MRI) data. However, these traditional techniques lack sensitivity and predictive value at the individual level. Machine-learning approaches have emerged as potential diagnostic and prognostic tools. We used an anatomically and spatially regularized support vector machine (SVM) framework to categorize schizophrenia and healthy individuals based on whole-brain gray matter densities estimated using voxel-based morphometry from structural MRI scans. The regularized SVM model yielded recognition accuracy of 86.6% in the training set of 127 individuals and validation accuracy of 83.5% in an independent set of 85 individuals. A sequential region-of-interest (ROI) selection step was adopted for feature selection, improving recognition accuracy to 92.0% in the training set and 89.4% in the validation set. The combined model achieved 96.6% sensitivity and 74.1% specificity. Seven ROIs were identified as the optimal discriminatory subset: the occipital fusiform gyrus, middle frontal gyrus, pars opercularis of the inferior frontal gyrus, anterior superior temporal gyrus, superior frontal gyrus, left thalamus and left lateral ventricle. These findings demonstrate the utility of spatial and anatomical priors in SVM for neuroimaging analyses in conjunction with sequential ROI selection in the recognition of schizophrenia.

## Introduction

Schizophrenia is a complex psychiatric disorder characterized by hallucinations, delusions, emotional disturbances and cognitive dysfunction. The diagnosis of schizophrenia is primarily dependent on the clinician’s evaluation based on comprehensive history taking, mental state examination, additional laboratory investigations whenever needed and corroborative information from caregivers and previous medical records^1^. Throughout the years, extensive work has been dedicated to optimizing the description and classification of psychoses from early nosological frames of Kraepelin^2^, Bleuler^3^, to current diagnostic taxonomies of Diagnostic and Statistical Manual of Mental Disorders, Fifth Edition (DSM-V)^4^ and International Classification of Diseases, 10th revision (ICD-10)^5^. To date, there has been increasing interest in the identification of reliable, objective biomarkers such as utilizing neuroimaging data to supplement clinical efforts in enhancing the diagnostic accuracy of psychoses including schizophrenia^6–8^.

The development of neuroimaging techniques such as magnetic resonance imaging (MRI) has enabled the noninvasive *in vivo* examination of brain structure. To date, there is substantial evidence from neuroimaging research that has revealed a range of structural brain abnormalities implicated in schizophrenia^9–12^; some of which are present at early course of illness^13–15^, or even before disease onset in high-risk individuals^16–18^. These implicated brain structures include localized volumetric reductions in the prefrontal and temporal lobes, specifically in the superior temporal gyrus, inferior and medial temporal lobes, amygdala, hippocampus and surrounding hippocampal gyrus, and the enlargement of lateral ventricles^12,19–23^.

Although traditional univariate methods have been commonly used for neuroanatomical investigation in schizophrenia, there are several limitations. Region-of-interest (ROI) approaches are confined to *a priori* defined brain regions and are unable to locate widespread patterns of abnormalities across the brain. Voxel-wise whole-brain methods like Voxel-Based Morphometry (VBM) require brain averaging, are unable to detect individual deviations, and often yield effect sizes too small to allow useful conclusions to be drawn in individual cases^24^. Thus, while significant differences in brain structure and function have been drawn at the *group* level, these results have limited generalizability at the *individual* level^25^. For neuroimaging to be more applicable in a clinical setting, one must preferably be able to make inferences at the level of the individual. As such, in an effort to utilize neuroimaging as a clinical diagnostic and prognostic tool, research has led to refined statistical approaches such as machine learning (ML) algorithms for pattern classification^8,26,27^. Given the multivariate nature of ML approaches, these techniques allow for improved sensitivity to subtle and spatially distributed brain differences that would likely remain undetected with the use of conventional univariate methods^28^. Furthermore, based on an identified pattern of brain abnormalities, ML methods enable better discrimination on an individual basis^28,29^. Thus, ML methods have been increasingly thought to hold potential as an auxiliary tool with a higher level of clinical translation to aid in diagnoses, clinical decision making, and outcome prediction.

A variety of ML techniques have been applied in neuroimaging settings. Support Vector Machine (SVM) has emerged as one of the most popular ML methods used in neuroimaging context, as it is able to effectively handle high-dimensional data and provide good classification results, thus avoiding overfitting of the data^24,30^. The fundamental aim of SVM is to classify data points by maximizing the margin between classes in a high dimensional space^31,32^. In essence, an optimal classifier is constructed through a “training phase”, whereby key brain features are identified in order to distinguish between two groups (such as patients versus controls), which is then applied to categorize new, unseen data in the “testing phase”. To date, the combination of neuroimaging and SVM has been applied in several studies involving neurological and psychiatric conditions. Whilst earlier SVM studies have applied the “leave-one-out”^33^ and SVM-Recursive Feature Elimination (RFE) techniques^28^ in conjunction with volumetric-based neuroimaging analyses, Yoon *et al*.^34^ also utilized SVM pattern classification based on principal components of cortical thickness to distinguish between schizophrenia and healthy controls and found accuracy rates between 88.8–93.6%. However, a more recent study involving a larger cohort has found lower accuracy rate of 70.4%^35^, suggesting that classification approaches and sample size may contribute to variation in accuracy rates. Furthermore, it has been reported that addition of refined feature selection methods as seen in a comparison of the studies by Davatzikos *et al*.^33^ and later by Fan *et al*.^28^ and the customization of kernels can improve the performance of classifiers^27,36^. In applying SVM approaches to neuroimaging data, it is necessary to recognize that brain images are structured data governed by underlying anatomical organization. To this end, Cuingnet *et al*.^37^ introduced a framework that takes into account the spatial and anatomical information in brain images with the supplementation of a brain atlas. This particular approach has been found to produce optimal classification accuracies in the presence of noise and has been applied to classify patients with Alzheimer’s disease^37,38^, although to our current knowledge, this has not been applied to other patient populations such as those with schizophrenia.

In this study, we thus sought to apply SVM with customized anatomical and spatial kernels to classify schizophrenia patients and healthy controls using structural MRI brain scans in a relatively large sample set and validated the classification results with an independent sample. In addition, we investigated the utility of a sequential step involving the use of an ROI selection algorithm to localize an optimal subset of ROIs to efficiently classify schizophrenia patients and healthy controls.

## Results

Through application of the anatomically and spatially regularized SVM on the DARTEL-transformed structural MRIs for the full cerebrum, the training accuracy reached 86.6% in the classification of schizophrenia and healthy controls. The full cerebrum accuracy was found to be 83.5% in the validation model.

**Figure 1:**
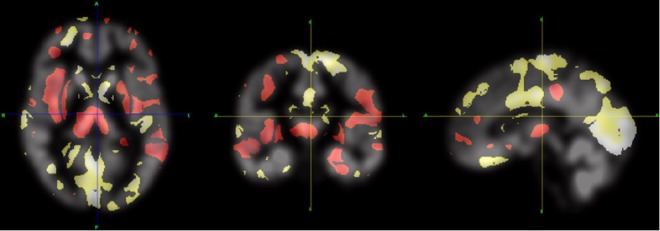
SVM coefficients (***w*** map). Regions in red represent areas of general increase in gray matter density. Regions in yellow represent areas of general decrease in gray matter density correlated to schizophrenia diagnosis.

**Figure 2:**
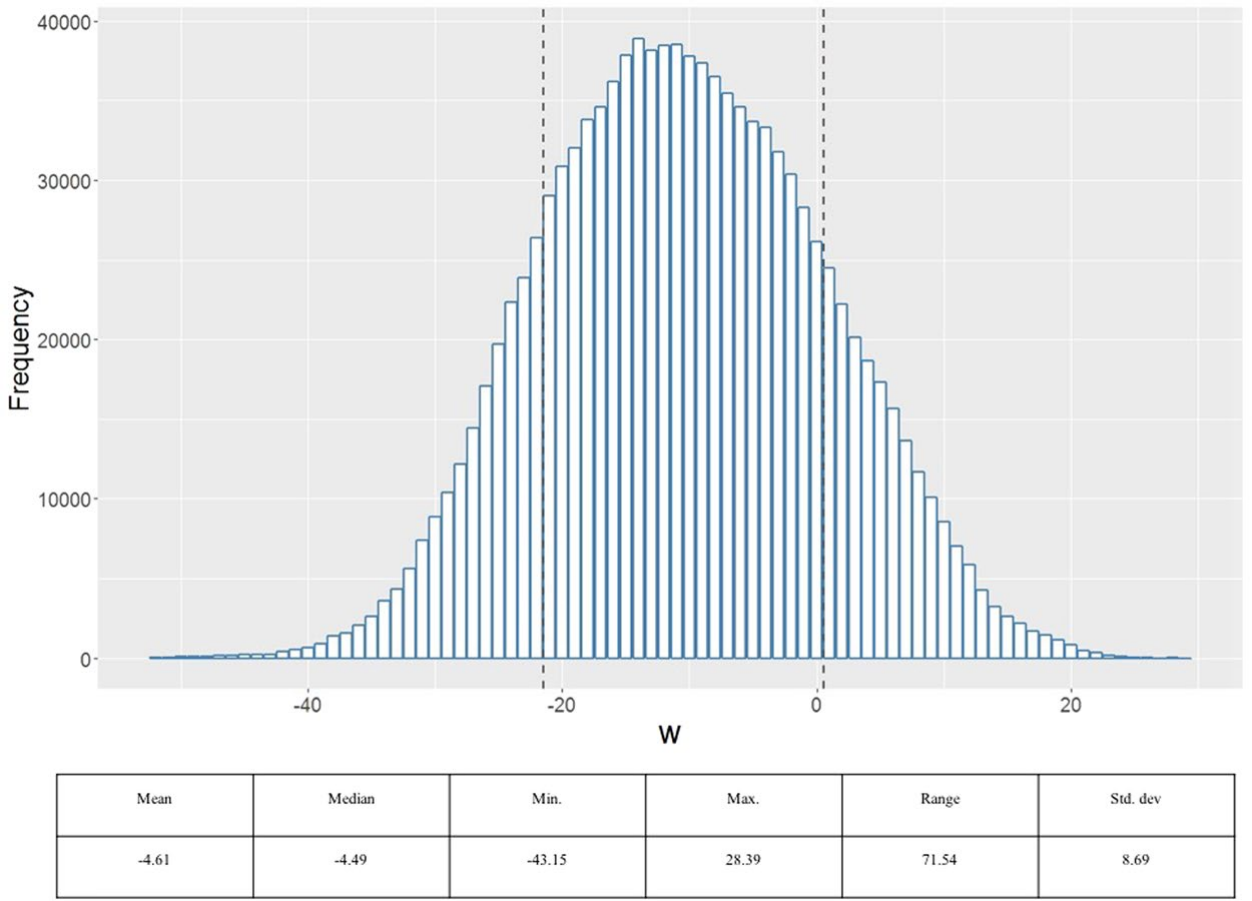
Distribution of SVM coefficients (***w*** distribution). The coefficients approximately follows a Gaussian distribution *N* (−4.61, 8.96^2^). Distributions of the coefficients in different ROIs are found to vary, indicating different levels of significance among all ROIs.

We found the 1.13 million entries of the coefficient vector (Fig. 1) approximately followed a Gaussian distribution ${\mathscr{N}}\,(\,-\,4.61,{8.69}^{2})$ (Fig. 2). Each element in the coefficient vector indicates the significance of an individual voxel in MRI classification derived by the underlying SVM model with the spatial and anatomical regularization. The red and yellow colors indicate association with general increases and decreases in gray matter densities respectively in schizophrenia patients compared to controls. For the positive coefficients, a higher intensity of the respective voxels was classified as schizophrenia whereas the opposite was true in the case of negative coefficients. The distributions of the coefficients within different ROIs were found to vary, indicating different levels of significance among all ROIs. Accordingly, we present a list detailing the distribution of weights corresponding to each ROI derived by solving the SVM model (Supplementary Table S3). This describes the proportion of positive and negative weight values as well as the mean (SD) weights with respect to voxels within each ROI.

**Figure 3:**
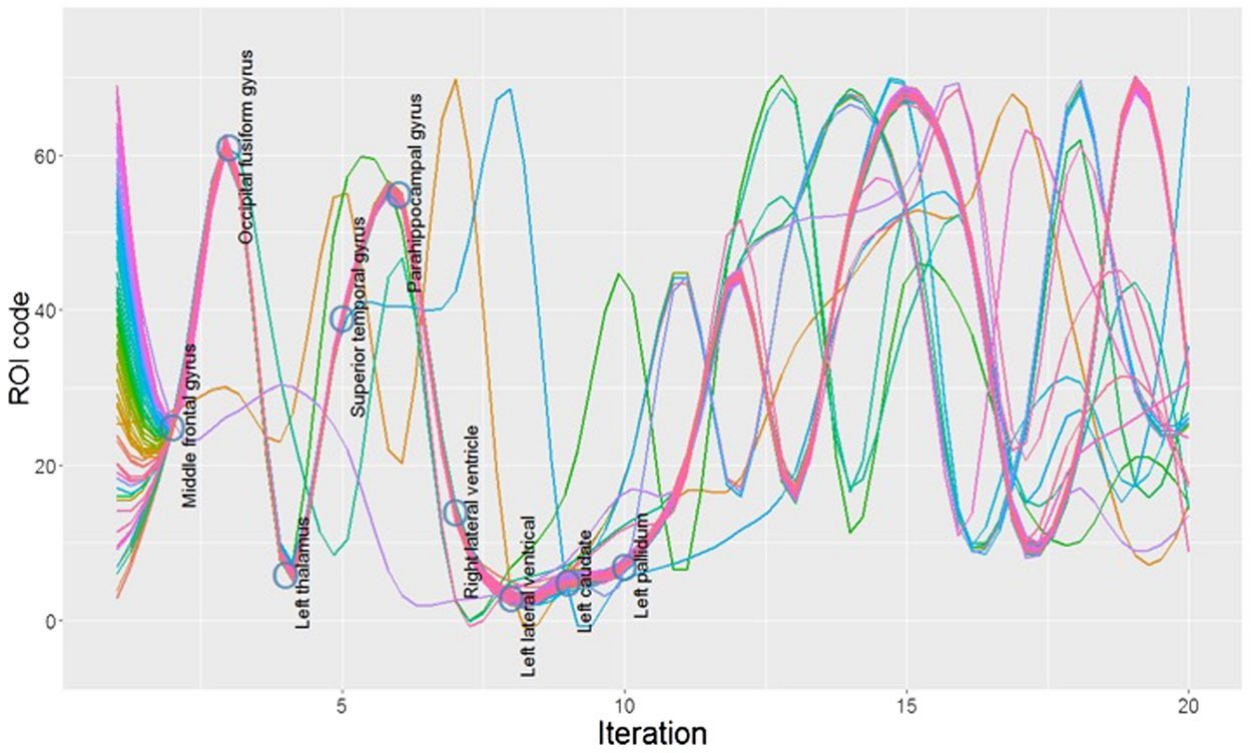
64 jittered smoothed selection paths with labeled ROIs. ROI IDs are further detailed in Table S3 in Supplementary Information.

**Figure 4:**
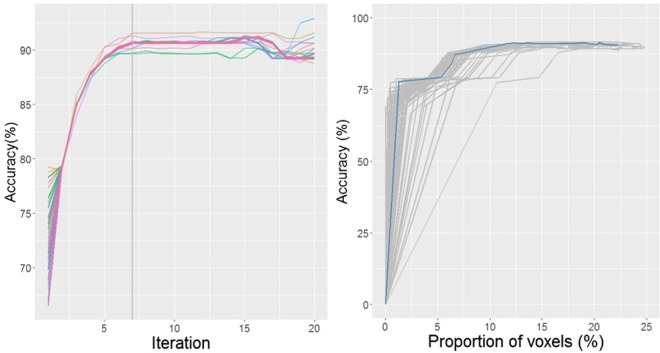
*Left:* Percentage (%) of accuracy per iteration for the 64 selection paths. *Right:* Trade-off between accuracy and voxels for the 64 selection paths.

In this study, there are a total of 64 ROIs under consideration. We selected a small subset of ROIs to predict schizophrenia for achieving the highest training accuracy using a proposed sequential ROI selection algorithm. We generated 64 different selection paths concerning the sequence of selected ROIs of interest as shown in the topmost graph in Fig. 3. Regardless of the choice of ROI at the initialization step in the algorithm, the derived selection paths appeared to have similar sequential patterns after several iterations. Specifically, the paths generated with different initial ROIs only shifted a small part of the corresponding selection paths while retaining the comparability for the rest in the path. In general, the common sequential pattern of all selection paths, which consists of several ROIs of interest, demonstrated the significance of the underlying ROIs in the classification. For example, as shown in Fig. 3, as to the underlying 64 selection paths, the most common ROIs generated by the algorithm from the 2nd to 5th iterations included the middle frontal gyrus, occipital fusiform gyrus, left putamen and superior parietal lobule. In terms of trade-off between the number of voxels and training accuracy, using more voxels in training generally increases training accuracy but on the other hand, reduces the efficiency in training phase. Correspondingly, the graphs in Fig. 4 depict the tradeoff between accuracy and voxel inclusion for the 64 selection paths.

The training accuracies of different initialized ROIs varied although after the first iteration all paths tended to converge in accuracy. The top five significant ROIs in terms of accuracy included the middle frontal gyrus (79.5%), precentral gyrus (79.3%), pars opercularis of the inferior frontal gyrus (79.0%), superior division of the lateral occipital cortex (78.2%) and posterior division of the supramarginal gyrus (78.1%). At each iteration, the next significant ROI was added into the model. The dynamic accuracy of each path was found to reach a turning point at the 7th iteration, indicating 7 as the optimal number of ROIs in the subset. Before the 7th iteration, the SVM was found to be under fitted with limited voxels as the input features. After the 7th iteration, the additional ROIs did not increase the training accuracy and there was a decrease in overall accuracy of the model. We provide a comprehensive list of the top 20 ROIs with the highest accuracy reached at the 7^th^ iteration in Supplementary Table S2. The 7 ROIs that were found to make up the optimal subset were the occipital fusiform gyrus, middle frontal gyrus, pars opercularis of the inferior frontal gyrus, anterior division of the superior temporal gyrus, left thalamus and left lateral ventricle. The 7 ROIs constituted 12.4% of total brain volume but yielded 92.0% in terms of training accuracy.

**Figure 5:**
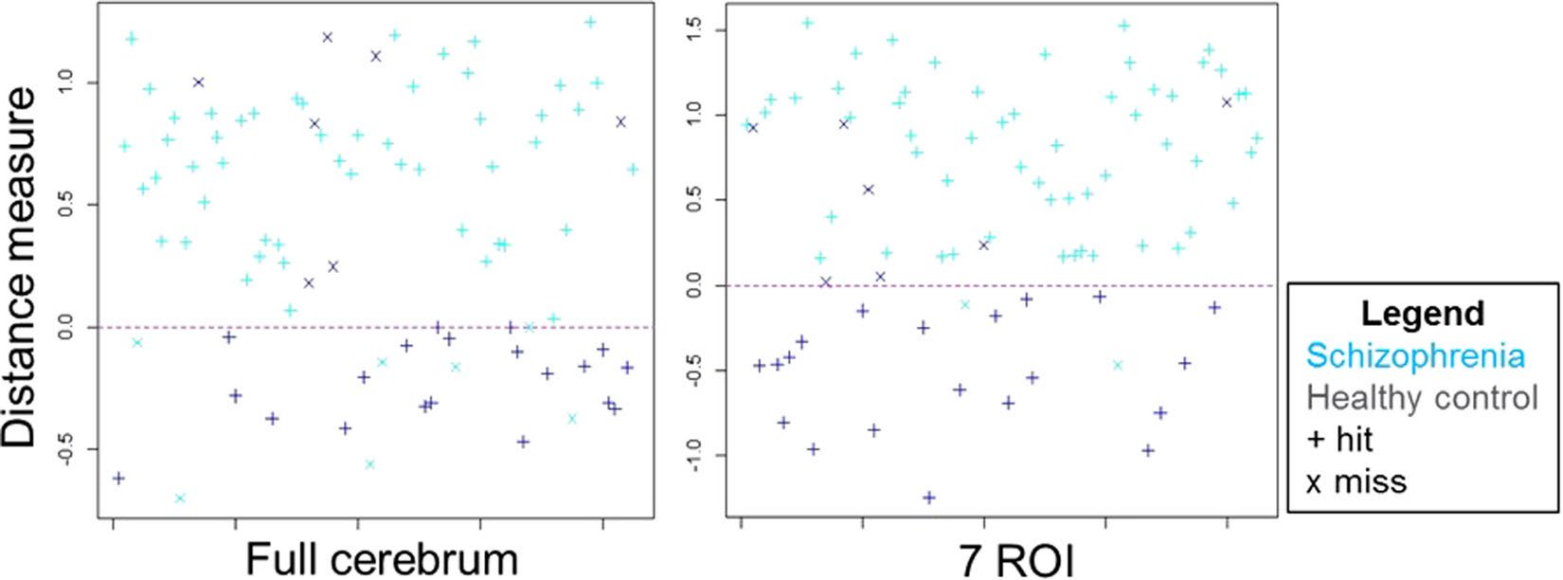
Distance maps of both full cerebrum and 7-ROI models on the validation group.

Within the optimal subset, the accuracies and the cumulative proportions of the 7 ROIs to the cerebrum volume were found to be: the occipital fusiform gyrus (77.5%, 1.3%); middle frontal gyrus (79.5%, 5.3%); pars opercularis of the inferior frontal gyrus (84.2%, 6.3%); anterior division of the superior temporal gyrus (87.2%, 6.7%); superior frontal gyrus (90.1%, 10.7%); left thalamus (91.1%, 11.6%) and left lateral ventricle (92.0%, 12.4%). Supplementary Table S1 highlights these 7 ROIs and presents their corresponding weight values in bold. After implementation of the sequential ROI selection step, the 7-ROI model achieved 89.4% classification accuracy in the validation set, 5.9% higher than the full cerebrum model. Furthermore, with regard to sensitivity (96.6% vs.87.9%), PPV (88.9% vs. 87.9%) and NPV (90.9% vs. 74.1%), the 7-ROI model was found to surpass the full cerebrum model in overall performance (Fig. 5 and Table 1).

**Table 1:** Comparison of model performance for full cerebrum and 7-ROI models (ACC: accuracy, SST: sensitivity, SPC: specificity, PPV: positive prediction value, NPV: negative prediction value).

Model	ACC (%)	SST (%)	SPC (%)	PPV (%)	NPV (%)
Full cerebrum	83.5	87.9	74.1	87.9	74.1
7 ROI	89.4	96.6	74.1	88.9	90.9

## Discussion

In the current study, we employed SVM with the spatial and anatomical regularization to create a data-driven model classifying SZ patients and HC based on key neuroanatomical features in structural MRI scans. The spatial proximity is encoded using the image connectivity as a regularization graph. Specifically, in the 3-dimensional image under consideration, the 6-connectivity graph is in terms of 6 face-connections for each voxel^37^. These 6 connected voxels are the 6 nearest neighbouring voxels. The spatial regularization uses the heat kernel on the graph^37^ to mute local variations, such as noise from the MRI scanner. The anatomical regularization counteracts the intensity interferences among different ROIs, so that each ROI is taken as a relatively independent input variable for SVM. This method effectively reduces overfitting caused by local variation in brain images and allows for spatial and anatomically coherent discrimination patterns to be derived, thereby increasing translational relevance and interpretation. In our study, we found that the regularized SVM model yielded recognition accuracy of 86.6% in the training set and subsequent validation accuracy of 83.5% in an independent group of subjects. The addition of sequential ROI selection improved the recognition accuracy of schizophrenia to 92% in the training set and 89.4% in the validation set. A subset of 7 ROIs selected was thus found to surpass the full cerebrum model in overall classification performance.

Pattern recognition studies of high dimensional data using machine learning methods have generally utilized two major approaches, i.e., the pre-model selection and post-model selection methods. The pre-model selection step utilizes machine learning models to determine the feature input for SVM such as principal component analysis (PCA) and independent component analysis (ICA)^39–41^ and the post-model selection adopts the coefficients of SVM as the significance measure to identify key features^33,35,42^. Unlike using ROIs (i.e., the sets of certain voxels of interest) in feature selection, the results derived by PCA or SVM are associated with discrete voxels, which might not necessarily be clinically interpretable. Specifically, we treated ROIs as independent variables in feature selection and analyzed the associated sets of the corresponding weights in this study. We used the sequential ROI selection process and took the training accuracy as the validation measure. The robustness of the sequential ROI selection process also ensures its generalizability in utility to other MRI studies. As shown in Fig. 5, the sequential ROI selection algorithm yielded 64 selection paths with different initial ROIs which approximately converge to a common sequential pattern.

Notably, the 89.4% classification accuracy obtained in the validation set with the additional application of the sequential ROI selection step appeared to be the highest, compared to relevant studies in literature^28,33,35^ which focused on voxel-based gray matter deficits in schizophrenia without incorporating both spatial and anatomical priors. In the first classification study which applied SVM to structural MRI data, Davatzikos *et al*.^33^ obtained an overall recognition accuracy of 81.1% using a “leave-one-out” method whereby the classifier was trained on all but one participant and later applied to test the left-out sample for cross-validation. These results provided an approximation of how generalizable the classifier would be to an independent cohort. Subsequently, Fan *et al*.^28^ used a SVM-Recursive Feature Elimination technique and observed an improved accuracy of 91.8% and 90.8% in distinguishing between the two groups of 69 SZ patients and 69 healthy controls. However, to the best of our knowledge, only two studies of schizophrenia validated their recognition models against an independent sample^35,43^. In the study conducted by Kawasaki and colleagues^43^, the classification accuracy of the training sample (60 subjects) was 75%, whereas the validation sample (32 subjects) achieved 80% accuracy. Nieuwenhuis *et al*.^35^ argued that the increase in accuracy derived from the validation sample could be partly due to the small sample size. Moreover, the study utilized a multivariate linear model instead of SVM. In the largest study of pattern recognition in schizophrenia to date, Nieuwenhuis and colleagues^35^ tested a SVM classifier developed with a sample of 239 subjects (SZ 53.6%) on a test group of 277 other participants (SZ 56.0%) and achieved accuracy of 71.4% and 70.4% respectively. With the addition of a 10% feature reduction step, the classification accuracy for the training set was improved to 86.8%, although accuracy of the validation set remained at 69.1%. One possible explanation for the reduction in recognition accuracy with larger sample sets could be attributed to greater variability in brain endophenotypes with sample size increase. While the above studies examined different cohorts of participants using different approaches, the method adopted in this study seems to be promising in recognition of schizophrenia by incorporating rich information in the SVM model.

With regard to structural brain abnormalities, the present analyses yielded a 7-ROI optimal model comprising of the occipital fusiform gyrus, middle frontal gyrus, pars opercularis of the inferior frontal gyrus, anterior superior temporal gyrus, superior frontal gyrus, left thalamus and left lateral ventricle. Our findings of implicated brain regions as well as the discriminative patterns derived from our model show consistency with that of extant voxel-based meta-analyses^12,23^ and prior classification studies^28,33,35,43–45^ in schizophrenia. For instance, we report decreased gray matter densities in frontal and superior temporal regions that corroborate with a vast body of literature showing volumetric reductions in these corresponding brain areas^12,23,46–48^. These reductions in frontotemporal brain regions are thought to underlie a range of cognitive deficits and clinical features seen in schizophrenia. Specifically, smaller inferior frontal gyrus volume has been found to correlate with greater negative symptoms, decreased motivation, as well as poorer language functioning in schizophrenia^49,50^. Medial prefrontal cortex reductions have been associated with deficits in executive functioning such as decision-making, computation, motor planning and imagery, discrimination, and reasoning^51^, as well as possible disruptions in the prefrontal-cingulate circuitry associated with non-goal directed and stimulus-independent processes^52^. The superior frontal gyrus, frequently found to be reduced in first-episode and neuroleptic naïve schizophrenia patients^16,53^, has been linked to anomalies in self-awareness, social cognition, and emotion^54,55^. Similarly, the superior temporal gyrus (STG) and subcortical regions such as the thalamus have been found to be implicated early in the illness course^56–59^. Correspondingly, our analyses showed decreased gray matter densities in these brain areas. Reduced STG and thalamic volume have been associated with positive symptoms including auditory hallucinations and thought disorder^60,61^, as well as deficits in working memory and attention^62,63^. Although less extensively examined, we found regional density decrease within the occipital cortex that has also been obtained in previous SVM classification studies^33,44^ which reported gray matter volumetric and concentration reductions, demonstrating the sensitivity of the SVM approach in detecting subtle brain regions that are altered in schizophrenia. Lastly, lateral ventricular enlargement presents as one of the earliest neuropathological hallmarks discovered in schizophrenia that has proven to be a robust finding across various cross-sectional and longitudinal studies^14,64,65^.

There are several limitations on this study. First, a pertinent issue is the sample size which can markedly affect diagnostic performance of the model. A limited number of samples can result in model overfitting and poor generalization of the method to independent data sets. As most of the existing studies have been carried out on a limited number of participants, it is difficult to draw definite conclusions and early results may not generalize well to other patient groups. Moreover, to circumvent the issue of small sample size, most studies have employed cross-validation frameworks by partitioning the data. However, it must be highlighted that the use of these techniques must be interpreted with caution as there is a serious risk of biasing classifier’s performance, particularly in the instance where data samples in the validation set are also present in the testing set^24^.

Second, although the SVM method has generally been found to provide better classification^32^, it is difficult to directly compare classification outcomes and ML methods across such studies due to the differences in clinical characteristics of the sample sets, image processing and classification pipelines. For example, Zarogianni and colleagues^24^ observed that ML methods, when performed in first-episode schizophrenia sample groups, produced poorer diagnostic performance compared to studies that included schizophrenia patients with greater chronicity of their illness^34,66,67^. It was hypothesized that this could be due to less pronounced and discernible neural alterations in the early onset group compared to chronic schizophrenia, thus influencing the accuracy of classifiers. Third, the presence of co-morbid disorders may also affect the sensitivity of the model in discriminating disease-specific patterns. Specifically, in a SVM classification study of early onset schizophrenia patients which included those with co-morbid substance use disorders, Zanetti and colleagues^67^ observed classification accuracy of 73.4% when compared against HC.

Future studies may want to consider pooling of subjects across sites^68^ to further increase the sample size of training and validation sets. Furthermore, multi-site studies may encompass more diverse, heterogeneous clinical populations, demonstrating a range of clinical manifestations related to a particular disorder^69^. In addition, further efforts are needed to examine the feasibility of using classification approaches to differentiate between psychiatric disorders. To aid classification performance, integrating neuroimaging indices with other biological markers such as genetic information may improve diagnoses, prediction and monitoring of treatment response and illness prognoses.

## Conclusion

We first used a SVM approach with customized anatomical and spatial kernels in the classification of patients with schizophrenia from healthy controls based on whole-brain gray matter volumetric data. We then optimized the SVM model by using a sequential ROI selection algorithm which identified an optimal 7-ROI set with a training accuracy of 92.0% and classification accuracy of 89.4% in an independent validation data set. The 7-ROI subset included the occipital fusiform gyrus, middle frontal gyrus, pars opercularis of the inferior frontal gyrus, anterior superior temporal gyrus, superior frontal gyrus, left thalamus and left lateral ventricle. This may potentially augment or supplement clinical approaches in assessing the clinical presentations of different individuals with schizophrenia which may be varied, complex and often recurring and relapsing.

## Methods

### Participants

A total of 212 participants were recruited, comprising of 141 patients with DSM-IV diagnosis of schizophrenia (SZ) (97 males and 44 females) and 71 healthy controls (HC) (45 males and 26 females). Written, informed consent was obtained from all participants after a thorough explanation of the study procedures. Diagnoses were made by the treating psychiatrist using information obtained from clinical history, existing medical records, interviews with the patient and significant others and the administration of the Structured Clinical Interview for DSM-IV Axis I disorders - Patient Edition (SCID-I/P)^70^. All patients were on a stable dose of antipsychotic medication for at least 2 weeks prior to recruitment and none had medication withdrawn for the purpose of the study. Participants with history of neurological illness or a diagnosis of alcohol or drug misuse in the preceding 3 months based on DSM-IV criteria were excluded. The SCID Non-Patient Edition (SCID-I/NP) was administered to healthy controls (HC) to rule out the presence of any other Axis I psychiatric disorder^71^. Handedness was determined with the administration of the Modified Edinburgh Questionnaire. This study was approved by the Institutional Review Board of the Institute of Mental Health, Singapore and the National Neuroscience Institute, Singapore. All procedures were carried out in accordance with the relevant guidelines and regulations outlined by the approving institutions.

Data from the 212 participants were divided into a training set consisting of 127 participants (60%) and validation set with 85 participants (40%). As to the partitioning of the data, the training and test sets were randomly selected by matching participants based on variables like age, gender and diagnosis listed in Table 2. The training set was used to train the SVM model to derive key anatomical regions and the model was then tested in the validation set. The permutation test was conducted for cross validation.

**Table 2:** Demographics and clinical characteristics of participants.

	Training Set	Testing Set	Total	p-value (t-test/chi-sq test)
*n*	127	85	212	
SCHZ/HC (SCHZ%)	84/43 (66.1%)	57/28 (67%)	141/72 (66.5%)	1
Male/Female (Male%)	85/42 (66.9%)	57/28 (67%)	142/70 (70.0%)	1
Age (Mean ± S.D)	41.0 ± 9.9	41.3 ± 9.4	41.1 ± 9.7	0.65
Handedness: Right/Left/Ambidextrous (Right%)	118/9 (92.9%)	75/9/1 (88.2%)	193/18/1 (91.0%)	0.52
Illness Duration, in Years (Mean ± S.D)	7.43 ± 8.1	7.38 ± 6.6	7.41 ± 7.5	0.19
PANSS Positive Symptom Score (Mean ± S.D)	11.0 ± 3.8	9.82 ± 3.6	10.5 ± 3.8	0.10
PANSS Negative Symptom Score (Mean ± S.D)	9.20 ± 3.1	8.63 ± 3.0	8.97 ± 3.1	0.16
PANSS Total Score (Mean ± S.D)	40.0 ± 9.1	37.9 ± 7.1	39.7 ± 8.5	0.25
CPZ Equivalent Dosage, in mg (Mean ± S.D)	189.1 ± 180.8	200.4 ± 185.8	193.7 ± 182.3	0.86

### Neuroimaging Data Acquisition

Magnetic resonance imaging was performed at the National Neuroscience Institute, Singapore, using a 3-Tesla whole body MRI scanner (Philips Achieva, Philips Medical Systems, Eindhoven, The Netherlands) with a SENSE head coil. Whole-brain scans were then acquired with a high resolution T1-weighted Magnetization Prepared Rapid Gradient Recalled Echo (MP-RAGE) sequence (repetition time (TR) = 8.4 s; echo time (TE) = 3.3 ms; flip angle = 8°). Each T1-weighted volume consisted of 180 axial slices of 0.9 mm thickness with no gap (field of view (FOV) = 230 × 230 mm; acquisition matrix = 256 × 256 pixels). The stability and high signal to noise ratio (SNR) are important factors contributing to enhance the accuracy and sensitivity in the measurement system. A regular quality control procedure was adopted to ensure the stability of a high signal-to-noise ratio.

### Neuroimaging Data Analyses

An optimized VBM protocol^72^ was applied using Statistical Parametric Mapping (SPM8) (http://www.fil.ion.ucl.ac.uk/spm/). In summary, we (1) segmented individual T1-weighted images into gray matter (GM), white matter (WM), and cerebrospinal fluid (CSF); (2) created a study-specific template consisting of both healthy participants and patients using nonlinear DARTEL registration^73^; (3) registered each GM probability map to the customized template in Montreal Neurological Institute (MNI) space and performed tissue segmentation; (4) performed modulation by multiplying voxel values by the Jacobian determinants derived from the spatial normalization step; (5) applied smoothing on the normalized GM maps by an 8-mm isotropic Gaussian kernel. The voxel intensities of these blurred segments indicate local presence or concentration of gray matter and are henceforth referred to as gray matter densities.

The Harvard-Oxford cortical and subcortical atlases^74^ were combined to identify the anatomical structure for which each MRI voxel belongs to. Since only the cerebrum is the region of interest in schizophrenia brain classification, the brain stem was excluded from the subcortical atlas. In total, 1,135,246 voxels from 64 anatomical structures were included in the complete atlas.

### SVM with Regularization Classification

Support Vector Machine (SVM) as a supervised learning model has been widely used in pattern detection with high dimensional data. It detects boundary samples (support vectors) and compounds the features of the support vectors to derive key features in classification using *L*_1_-norm or *L*_2_-norm. For the problem under consideration, the features of the subjects used in training in the SVM model are individual voxels. The dimensions of the model are equal to the number of voxels in a single brain MRI scan. The value of the coefficient in the model associated with each voxel indicates the relative importance of the respect voxel in classifying subjects into two groups (i.e., SZ or HC).

In this study, we applied the framework of spatial and anatomical regularization of SVM developed by Cuingnet *et al*.^37^ to the classification of neuroimaging data for patients with schizophrenia, which extended its application to the classification of patients with Alzheimer’s disease discussed in^37^. By introducing the regularization operator to the SVM, the classification function is constrained to be smooth with respect to the spatial and anatomical priors. Briefly, the underlying regularization is defined based on spatial proximity and anatomical proximity using Laplacian operator. Specifically, the spatial regularization uses the heat kernel and 6-connectivity-model to mute the local variations, such as noise from MRI scanner. The anatomical regularization counteracts the intensity interferences among different ROIs. In previous applications using SVM, when the training set is small, a large radius of isotropic kernel is usually required. Then, the anatomical kernel would play a key role in retaining the key features of each anatomical structure. In general, the combination of both spatial and anatomical kernels could adjust the spatial and anatomical factors appropriately within the brain image. This framework effectively reduces the overfitting caused by local variation in brain images and allows for spatial and anatomically coherent discrimination patterns to be derived, resulting in increasing translational relevance and interpretation. A reader is referred to^37^ for a detailed description and explanation on the underlying SVM framework.

Let ${\mathscr{V}}$ denote the domain the 3D images and *v* denote an element of ${\mathscr{V}}$ (i.e., a voxel). Let $\chi ={L}^{2}({\mathscr{V}})$ represent the set of integral functions on ${\mathscr{V}}$ equipped with the canonical inner product denoted by $\langle .,.\rangle .$ Let *x*_s_ ∈ *χ* be the data of a given subject *s*. In this study, *x*_s_ can be viewed as an element of *R*^*m*^, where *m* denotes the total number of voxels since the images are discrete. Assume there is a group of *N* subjects with the corresponding data *x*_s_ ∈ *χ*, $s=1,\ldots ,\,N$. Each subject is associated with a group *y*_*s*_ ∈ {−1, 1} (e.g., diseased or healthy), $s=1,\ldots ,\,N$. In combination with these two regularization terms, the underlying SVM optimization problem was presented in Equation(21) of the ref.^37^ That is,

$${\min }_{{\boldsymbol{w}}\in {\boldsymbol{X}},b\in R}\frac{1}{N}{\sum }_{s=1}^{N}{\ell }_{hinge}({y}_{s}[\,\langle {\boldsymbol{w}},{x}_{s}\rangle +b])+\lambda (||{e}^{\frac{{\beta }_{s}}{2}{L}_{s}}{\boldsymbol{w}}|{|}^{2}+||{e}^{\frac{{\beta }_{a}}{2}{L}_{a}}{\boldsymbol{w}}|{|}^{2}),$$

where *λ* ∈ *R*^+^ is the regularization parameter and ${\ell }_{hinge}(\,\cdot \,)$ is the hinge loss function commonly used in SVM defined by ${\ell }_{hinge}(u):={(1-u)}^{+}$, *u* ∈ *R*. Here, *L*_*s*_ and *L*_*a*_ refer to the associated Laplacians of the graph encoding spatial proximity and anatomical proximity, respectively. *β*_*a*_ and *β*_*s*_ denote anatomical and spatial hyperparameters which control the spatial and anatomical effect in the regularization. Each element in the coefficient vector *w* = (*w*_*v*_)_*v*∈*V*_ corresponds to a voxel and indicates the significance of the individual voxel in MRI classification. *b* is the usual scalar parameter in defining the hyperplane in SVM. It is known from (37, Equation (22)) that the above minimization problem is equivalent to an SVM optimization problem with kernel

$${K}_{{\beta }_{a},{\beta }_{s}}({{\boldsymbol{x}}}_{1},{{\boldsymbol{x}}}_{2})={{\boldsymbol{x}}}_{1}^{T}{({e}^{{\beta }_{a}{L}_{a}}+{e}^{{\beta }_{s}{L}_{s}})}^{-1}{{\boldsymbol{x}}}_{2}\cdot $$

Mathematically, the underlying kernel function involves two items concerning matrix exponential associated with information pertaining to anatomical and spatial voxel adjacency. This offers an improved classification performance for problems under consideration. Generally, the kernel takes local voxel variance and cross-structural variance into account. It uses the space kernel to reduce the variance of neighboring voxels and the binary anatomical kernel to segregate the cerebrum according to the anatomical structure. In this study, the binary anatomical voxel adjacency matrix (*L*_*a*_) was derived from the Harvard-Oxford cortical and subcortical atlases associated with each voxel with the relevant structural label. The binary anatomical voxel adjacency matrix is a large sparse matrix with the dimension being equal to the number of pixels in an MRI scan. The binary value indicates whether two voxels are from the same anatomical structure or not. The probabilistic spatial voxel adjacency matrix (*L*_*s*_) was derived from the 6-connectivity model and the parameters *β*_*a*_, *β*_*s*_ and *λ* were determined by the grid search. The map in Fig. 1 presents a concept visualization to reflect how the effect of the high dimensional kernels would translate into a real cerebrum sample. With the anatomical and spatial regularization, the coefficients are well smoothed within the same anatomical structure and regularized the reciprocal effect among different ROIs.

### Sequential ROI Selection

In the machine learning framework, various learning strategies may be applied for feature selection. In general, the *L*_1_-norm performs well with the SVM algorithms in feature selection. However, in this study, we did not choose to use the *L*_1_-norm as it selects features by voxel, which might not have specific anatomical and clinical interpretation. Instead, we propose the use of a sequential ROI selection algorithm to identify the key ROIs.

To perform the sequential ROI selection, at the initialization step, we defined a singleton set of ROI by choosing an arbitrary ROI from 64 ROIs under consideration. This set was updated progressively by adding a new ROI from the remaining ROIs at next iteration. Here the newly added ROI was chosen based on the criteria that the training accuracy in predicting schizophrenia using this ROI together with ROIs chosen in previous iterations could achieve the highest accuracy, compared to training prediction accuracies attained by using any other individual remaining candidate and those ROIs selected previously. This process continues until the total number of ROIs of the sequential ROI selection is reached. Hence, we then derived a selection path of all ROIs (i.e., 64 ROIs) under consideration. The selection process is a greedy-forward algorithm in which an optimization problem is solved for choosing the optimal ROI at each step. In view of the influence of the initial ROI on the subsequent sequence of ROIs, we ran through 64 scenarios with each ROI being chosen as the initial ROI. In general, *n* selection paths were derived with *n* different initializations of individual ROIs and the corresponding training accuracy at each step in the selection paths were noted. Eventually, the more significant features, i.e., ROIs, in classification were included in the selected ROI training set. Due to the large number of voxels under consideration, the training predictions involved at each step are large scale problems, subsequently incurring massive computational efforts in implementation. To improve the efficiency of the sequential ROI selection process, it necessitates balancing both computational load and prediction accuracy. Specifically, we were interested to investigate the tradeoff between the voxels used and the training accuracy achieved. Thus, we chose a number of ROIs of interest which as small as possible (i.e., 7 ROIs) from all derived selection paths that resulted in the highest accuracy in prediction. Furthermore, the proposed selection process is in the context of a robust optimization approach which seeks to generate a relatively stable solution of interest, under the uncertainty of randomly selected training and test sets.

## Electronic supplementary material

Supplementary Material
