# Supplementary material for: Recognition of Schizophrenia with Regularized Support Vector Machine and Sequential Region of Interest Selection using Structural Magnetic Resonance Imaging
Source: Sci Rep. 2018 Sep 14;8:13858. doi: 10.1038/s41598-018-32290-9 (PMC6138658; doi:10.1038/s41598-018-32290-9)
Supplement: Supplementary file 1 — Supplementary Material [file 41598_2018_32290_MOESM1_ESM.docx]

**Title: Recognition of Schizophrenia with Regularized Support Vector Machine and Sequential Region of Interest Selection using Structural Magnetic Resonance Imaging**

**Authors:** Rowena **Chin**^†1^, MSc., Alex Xiaobin **You**^†2^, MSc., Fanwen **Meng**^†2^, Ph.D., Juan **Zhou**^3^, Ph.D., Kang **Sim**^*1,4^, MBBS, M.Med (Psychiatry), MScHPE, FAMS

^†^ *Co-first authors: These authors contributed equally to this work.*

^1^ Research Division, Institute of Mental Health, Singapore, 10 Buangkok View, Singapore 539747, Singapore

^2^ Health Services & Outcomes Research, National Healthcare Group, 3 Fusionopolis Link, Singapore 138543, Singapore

^3^ Neuroscience & Behavioral Disorders Program, Duke-NUS Medical School, 8 College Road, Singapore 169857, Singapore

^4^ Department of General Psychiatry, Institute of Mental Health/Woodbridge Hospital, Singapore, 10 Buangkok View, Singapore 539747, Singapore

**Supplementary Information**

| **ROI** | **Positive** | **Negative** | **Mean(Std.)** |
| --- | --- | --- | --- |
| **left lateral ventricle** | **46.6%** | **53.4%** | **-0.73 ( 6.29 )** |
| **left thalamus** | **32.1%** | **67.9%** | **-3.02 ( 6.13 )** |
| left caudate | 79.4% | 20.6% | 3.86 ( 5.86 ) |
| left putamen | 42.3% | 57.7% | -2.96 ( 10.4 ) |
| left pallidum | 67.7% | 32.3% | 2.5 ( 5.59 ) |
| left hippocampus | 0.2% | 99.8% | -9.09 ( 4.28 ) |
| left amygdala | 0.0% | 100.0% | -15.41 ( 3.39 ) |
| left accumbens | 0.0% | 100.0% | -7.51 ( 2.23 ) |
| right lateral ventricle | 42.8% | 57.2% | -1.91 ( 5.93 ) |
| right thalamus | 14.7% | 85.3% | -6.1 ( 5.75 ) |
| right caudate | 59.1% | 40.9% | 1.01 ( 7.06 ) |
| right putamen | 41.6% | 58.4% | -6.46 ( 14.67 ) |
| right pallidum | 96.1% | 3.9% | 5.41 ( 3.65 ) |
| right hippocampus | 0.1% | 99.9% | -10.94 ( 4.92 ) |
| right amygdala | 0.0% | 100.0% | -16.31 ( 2.99 ) |
| right accumbens | 0.2% | 99.8% | -11.06 ( 3.18 ) |
| frontal pole | 35.0% | 65.0% | -3.29 ( 7.68 ) |
| insular cortex | 22.7% | 77.3% | -7.09 ( 8.55 ) |
| **superior frontal gyrus** | **26.9%** | **73.1%** | **-3.16 ( 5.59 )** |
| **middle frontal gyrus** | **26.6%** | **73.4%** | **-4.32 ( 6.5 )** |
| inferior frontal gyrus; pars triangularis | 12.1% | 87.9% | -7.03 ( 5.96 ) |
| **inferior frontal gyrus; pars opercularis** | **6.0%** | **94.0%** | **-13.05 ( 9.73 )** |
| precentral gyrus | 22.5% | 77.5% | -5.17 ( 7.26 ) |
| temporal pole | 13.6% | 86.4% | -8.03 ( 7 ) |
| **superior temporal gyrus; anterior division** | **8.0%** | **92.0%** | **-12.27 ( 8.48 )** |
| superior temporal gyrus; posterior division | 9.1% | 90.9% | -10.43 ( 7.71 ) |
| middle temporal gyrus; anterior division | 19.0% | 81.0% | -8.01 ( 8.15 ) |
| middle temporal gyrus; posterior division | 15.2% | 84.8% | -9.93 ( 9.35 ) |
| middle temporal gyrus; temporooccipital part | 12.0% | 88.0% | -9.69 ( 8.34 ) |
| inferior temporal gyrus; anterior division | 7.7% | 92.3% | -9.36 ( 5.11 ) |
| inferior temporal gyrus; posterior division | 34.1% | 65.9% | -4.39 ( 9.34 ) |
| inferior temporal gyrus; temporooccipital part | 10.8% | 89.2% | -8.99 ( 7.2 ) |
| postcentral gyrus | 22.2% | 77.8% | -5.81 ( 7.02 ) |
| superior parietal lobule | 19.0% | 81.0% | -5.19 ( 5.8 ) |
| supramarginal gyrus; anterior division | 38.1% | 61.9% | -2.65 ( 7.61 ) |
| supramarginal gyrus; posterior division | 5.2% | 94.8% | -8.74 ( 5.01 ) |
| angular gyrus | 25.8% | 74.2% | -5.3 ( 7.07 ) |
| lateral occipital cortex; superior division | 36.8% | 63.2% | -1.56 ( 8.59 ) |
| lateral occipital cortex; inferior division | 36.3% | 63.7% | -3.21 ( 7.59 ) |
| intracalcarine cortex | 42.4% | 57.6% | -0.76 ( 6.73 ) |
| frontal medial cortex | 6.5% | 93.5% | -11 ( 7.31 ) |
| juxtapositional lobule cortex | 23.6% | 76.4% | -4.86 ( 6.44 ) |
| subcallosal cortex | 9.6% | 90.4% | -9.78 ( 6.28 ) |
| paracingulate gyrus | 12.8% | 87.2% | -7.28 ( 6.17 ) |
| cingulate gyrus; anterior division | 21.9% | 78.1% | -4.22 ( 4.91 ) |
| cingulate gyrus; posterior division | 15.9% | 84.1% | -4.73 ( 4.88 ) |
| precuneous cortex | 54.3% | 45.7% | 1.12 ( 5.94 ) |
| cuneal cortex | 58.7% | 41.3% | 0.32 ( 8.87 ) |
| frontal orbital cortex | 18.6% | 81.4% | -8.27 ( 9.12 ) |
| parahippocampal gyrus; anterior division | 30.0% | 70.0% | -3.81 ( 7.28 ) |
| parahippocampal gyrus; posterior division | 12.6% | 87.4% | -7.28 ( 6.77 ) |
| lingual gyrus | 47.8% | 52.2% | 0.08 ( 7.28 ) |
| temporal fusiform cortex; anterior division | 4.1% | 95.9% | -9.97 ( 4.68 ) |
| temporal fusiform cortex; posterior division | 8.7% | 91.3% | -9.3 ( 6.92 ) |
| temporal occipital fusiform cortex | 4.7% | 95.3% | -10.35 ( 5.24 ) |
| **occipital fusiform gyrus** | **14.6%** | **85.4%** | **-9.15 ( 10.06 )** |
| frontal operculum cortex | 3.3% | 96.7% | -17.53 ( 7.52 ) |
| central opercular cortex | 8.4% | 91.6% | -13.3 ( 9.05 ) |
| parietal operculum cortex | 21.6% | 78.4% | -6.92 ( 7.57 ) |
| planum polare | 0.1% | 99.9% | -15.03 ( 7.1 ) |
| heschls gyrus | 7.1% | 92.9% | -11.17 ( 7.96 ) |
| planum temporale | 17.9% | 82.1% | -8.3 ( 7.96 ) |
| supracalcarine cortex | 1.9% | 98.1% | -10.39 ( 5.7 ) |
| occipital pole | 16.7% | 83.3% | -6.09 ( 6.42 ) |

**Supplementary Table S1.** Proportion of positive and negative weights and mean weights corresponding to voxels within ROIs.

| Iteration | New ROI added | Accuracy (%) | Cumulative volume (% of the whole cerebrum) |
| --- | --- | --- | --- |
| 1 | occipital fusiform gyrus | 77.51 | 1.28 |
| 2 | middle frontal gyrus | 79.47 | 5.29 |
| 3 | inferior frontal gyrus; pars opercularis | 84.15 | 6.32 |
| 4 | superior temporal gyrus; anterior division | 87.23 | 6.71 |
| 5 | superior frontal gyrus | 90.06 | 10.70 |
| 6 | left thalamus | 91.14 | 11.61 |
| 7 | left lateral ventricle | 92.04 | 12.43 |
| 8 | left caudate | 91.25 | 12.78 |
| 9 | left pallidum | 91.51 | 12.97 |
| 10 | right caudate | 90.89 | 13.33 |
| 11 | right pallidum | 91.48 | 13.52 |
| 12 | intracalcarine cortex | 91.49 | 14.51 |
| 13 | precuneous cortex | 91.18 | 18.46 |
| 14 | cuneal cortex | 91.34 | 19.33 |
| 15 | parahippocampal gyrus; anterior division | 90.45 | 20.21 |
| 16 | supracalcarine cortex | 90.95 | 20.40 |
| 17 | left acumens | 91.64 | 20.46 |
| 18 | right putamen | 91.28 | 21.03 |
| 19 | left hippocampus | 90.34 | 21.51 |
| 20 | right lateral ventricle | 90.72 | 22.23 |

**Supplementary Table S2.** Top 20 ROIs in the optimal selection path with the highest accuracy reached at 7^th^ iteration.

| **ID** | **ROI name** | **Volume (mm^3^)** | **Volume (% of the whole cerebrum)** | **x** | **y** | **z** |
| --- | --- | --- | --- | --- | --- | --- |
| 3 | left lateral ventricle | 9,298 | 0.82 | 79.6 | 138.4 | 87.1 |
| 4 | left thalamus | 10,385 | 0.91 | 82.2 | 135.8 | 79.2 |
| 5 | left caudate | 3,949 | 0.35 | 79.3 | 138.7 | 82.7 |
| 6 | left putamen | 6,397 | 0.56 | 67.1 | 150.9 | 73.4 |
| 7 | left pallidum | 2,133 | 0.19 | 72.7 | 145.3 | 71.8 |
| 9 | left hippocampus | 5,451 | 0.48 | 67.0 | 151.0 | 58.8 |
| 10 | left amygdala | 2,391 | 0.21 | 69.3 | 148.7 | 55.1 |
| 11 | left accumbens | 756 | 0.07 | 82.5 | 135.5 | 65.9 |
| 14 | right lateral ventricle | 8,282 | 0.73 | 105.3 | 112.7 | 87.2 |
| 15 | right thalamus | 10,211 | 0.90 | 102.8 | 115.2 | 79.7 |
| 16 | right caudate | 4,127 | 0.36 | 105.3 | 112.7 | 83.4 |
| 17 | right putamen | 6,397 | 0.56 | 117.5 | 100.5 | 73.3 |
| 18 | right pallidum | 2,118 | 0.19 | 111.9 | 106.1 | 71.8 |
| 19 | right hippocampus | 5,563 | 0.49 | 118.5 | 99.5 | 58.7 |
| 20 | right amygdala | 2,697 | 0.24 | 115.1 | 102.9 | 55.3 |
| 21 | right accumbens | 666 | 0.06 | 101.4 | 116.6 | 66.6 |
| 22 | frontal pole | 120,948 | 10.65 | 94.6 | 123.4 | 80.9 |
| 23 | insular cortex | 21,449 | 1.89 | 92.8 | 125.2 | 73.0 |
| 24 | superior frontal gyrus | 45,246 | 3.99 | 91.8 | 126.2 | 129.4 |
| 25 | middle frontal gyrus | 45,499 | 4.01 | 91.4 | 126.6 | 115.4 |
| 26 | inferior frontal gyrus; pars triangularis | 9,503 | 0.84 | 88.3 | 129.7 | 81.2 |
| 27 | inferior frontal gyrus; pars opercularis | 11,674 | 1.03 | 89.9 | 128.1 | 88.8 |
| 28 | precentral gyrus | 70,031 | 6.17 | 91.9 | 126.1 | 122.8 |
| 29 | temporal pole | 37,827 | 3.33 | 92.2 | 125.8 | 43.4 |
| 30 | superior temporal gyrus; anterior division | 4,482 | 0.39 | 92.8 | 125.2 | 63.9 |
| 31 | superior temporal gyrus; posterior division | 6,488 | 0.57 | 91.9 | 126.1 | 75.7 |
| 32 | middle temporal gyrus; anterior division | 6,952 | 0.61 | 89.9 | 128.1 | 49.7 |
| 33 | middle temporal gyrus; posterior division | 21,879 | 1.93 | 92.1 | 125.9 | 61.4 |
| 34 | middle temporal gyrus; temporooccipital part | 16,041 | 1.41 | 101.0 | 117.0 | 74.3 |
| 35 | inferior temporal gyrus; anterior division | 5,385 | 0.47 | 90.7 | 127.3 | 32.9 |
| 36 | inferior temporal gyrus; posterior division | 15,745 | 1.39 | 90.3 | 127.7 | 46.0 |
| 37 | inferior temporal gyrus; temporooccipital part | 11,973 | 1.05 | 96.0 | 122.0 | 56.4 |
| 38 | postcentral gyrus | 55,269 | 4.87 | 89.3 | 128.7 | 125.1 |
| 39 | superior parietal lobule | 23,450 | 2.07 | 92.1 | 125.9 | 131.2 |
| 40 | supramarginal gyrus; anterior division | 13,831 | 1.22 | 87.7 | 130.3 | 110.5 |
| 41 | supramarginal gyrus; posterior division | 18,720 | 1.65 | 95.9 | 122.1 | 106.4 |
| 42 | angular gyrus | 19,542 | 1.72 | 102.9 | 115.1 | 104.3 |
| 43 | lateral occipital cortex; superior division | 78,223 | 6.89 | 92.0 | 126.0 | 111.4 |
| 44 | lateral occipital cortex; inferior division | 32,849 | 2.89 | 92.2 | 125.8 | 71.3 |
| 45 | intracalcarine cortex | 11,317 | 1.00 | 93.5 | 124.5 | 81.2 |
| 46 | frontal medial cortex | 7,796 | 0.69 | 92.2 | 125.8 | 54.5 |
| 47 | juxtapositional lobule cortex | 11,954 | 1.05 | 92.5 | 125.5 | 129.9 |
| 48 | subcallosal cortex | 9,021 | 0.79 | 91.9 | 126.1 | 58.2 |
| 49 | paracingulate gyrus | 23,252 | 2.05 | 92.3 | 125.7 | 94.8 |
| 50 | cingulate gyrus; anterior division | 20,844 | 1.84 | 92.8 | 125.2 | 97.3 |
| 51 | cingulate gyrus; posterior division | 19,228 | 1.69 | 92.8 | 125.2 | 103.0 |
| 52 | precuneous cortex | 44,787 | 3.95 | 93.0 | 125.0 | 111.0 |
| 53 | cuneal cortex | 9,959 | 0.88 | 93.2 | 124.8 | 100.5 |
| 54 | frontal orbital cortex | 25,157 | 2.22 | 89.5 | 128.5 | 56.6 |
| 55 | parahippocampal gyrus; anterior division | 9,956 | 0.88 | 93.5 | 124.5 | 42.7 |
| 56 | parahippocampal gyrus; posterior division | 5,698 | 0.50 | 90.6 | 127.4 | 56.2 |
| 57 | lingual gyrus | 26,880 | 2.37 | 93.4 | 124.6 | 67.8 |
| 58 | temporal fusiform cortex; anterior division | 4,901 | 0.43 | 89.9 | 128.1 | 30.9 |
| 59 | temporal fusiform cortex; posterior division | 12,680 | 1.12 | 88.6 | 129.4 | 46.7 |
| 60 | temporal occipital fusiform cortex | 11,759 | 1.04 | 97.0 | 121.0 | 56.7 |
| 61 | occipital fusiform gyrus | 14,538 | 1.28 | 91.9 | 126.1 | 60.0 |
| 62 | frontal operculum cortex | 5,313 | 0.47 | 90.2 | 127.8 | 77.7 |
| 63 | central opercular cortex | 14,953 | 1.32 | 90.6 | 127.4 | 84.5 |
| 64 | parietal operculum cortex | 8,739 | 0.77 | 91.4 | 126.6 | 94.0 |
| 65 | planum polare | 5,871 | 0.52 | 93.7 | 124.3 | 65.7 |
| 66 | heschls gyrus | 4,730 | 0.42 | 89.7 | 128.3 | 80.1 |
| 67 | planum temporale | 8,032 | 0.71 | 86.7 | 131.3 | 84.4 |
| 68 | supracalcarine cortex | 2,081 | 0.18 | 94.1 | 123.9 | 87.2 |
| 69 | occipital pole | 41,973 | 3.70 | 92.0 | 126.0 | 80.5 |

**Supplementary Table S3.** ROI IDs (ID 1-2, 12-13 are cerebral white matter and cortex, omitted) used in ROI selection, volume, percentage of the whole cerebrum and coordinates.

**Sequential Region of Interest Selection Algorithm**

**Step 0.** Initialization. Let $=\{1, 2, \ldots, K_{1}\}$ . Let $R_{k}, k \in I,$ denote the region of interest (ROI). Set $K_{1}=64, K_{2}=64, \hat{I}=\emptyset$.

**Step 1.** Choose an arbitrary $i\in I\backslash\{\hat{I}\}$. Define $I^{i}=\left\{ i \right\}.$

**Step 2.** Solve the following optimization problem

$$\max_{j\in I\backslash I^{i}} \rho_{i}$$

where, for $j\in I\backslash I^{i},$ $\rho_{j}$ denotes the training accuracy of predicting Schizophrenia using the following ROIs, $\left\{ R_{j,} R_{k,} k\in I^{i} \right\}.$ Let $j^{*}=\mathrm{argmax}\left\{ \rho_{j} \right| j\in I\backslash I^{i}\}.$ Update $I^{i}=I^{i}\cup j^{*}.$

**Step 3.** If $\left| I^{i} \right|\leq K_{2,}$ go to Step 2. Otherwise, update $\hat{I}=\left\{ i \right\}\cup\hat{I}.$ Go to Step 4.

**Step 4.** If $\left| \hat{I} \right|=K_{1}$, stop. Otherwise, go to Step 1.

**Supplementary Figure S1.** The proposed algorithm will generate 64 sequential ROI selection paths, each path consisting of 64 ROIs in order. In computational implementation, one may choose a smaller number of $K_{2}.$ For example, if choose $K_{2}=20$, each selection path will be of 20 ROIs. The choice of $K_{2}$ may vary and usually depends on considerations of computational efficiency and prediction accuracy with respect to the cohort under investigation.
